# DNA repair deficiency as circulating biomarker in prostate cancer

**DOI:** 10.3389/fonc.2023.1115241

**Published:** 2023-01-30

**Authors:** Martina Catalano, Daniele Generali, Marta Gatti, Barbara Riboli, Leda Paganini, Gabriella Nesi, Giandomenico Roviello

**Affiliations:** ^1^ School of Human Health Sciences, University of Florence, Florence, Italy; ^2^ Department of Medical, Surgical and Health Sciences, University of Trieste, Cattinara Hospital Trieste, Trieste, Italy; ^3^ Servizio di Citogenetica e Genetica - Azienda Socio-Sanitaria Territoriale (ASST) di Cremona, Cremona, Italy; ^4^ Department of Health Sciences, University of Florence, Florence, Italy

**Keywords:** DNA damage repair, prostate cancer, liquid biopsy, circulating biomarker, circulating tumor DNA

## Abstract

Deleterious aberrations in DNA repair genes are actionable in approximately 25% of metastatic castration-resistant prostate cancers (mCRPC) patients. Homology recombination repair (HRR) is the DNA damage repair (DDR) mechanism most frequently altered in prostate cancer; of note *BRCA2* is the most frequently altered DDR gene in this tumor. Poly ADP-ribose polymerase inhibitors showed antitumor activity with a improvement in overall survival in mCRPC carrying somatic and/or germline alterations of HHR. Germline mutations are tested on peripheral blood samples using DNA extracted from peripheral blood leukocytes, while the somatic alterations are assessed by extracting DNA from a tumor tissue sample. However, each of these genetic tests have some limitations: the somatic tests are related to the sample availability and tumor heterogeneity, while the germline testing are mainly related to the inability to detect somatic HRR mutations. Therefore, the liquid biopsy, a non-invasive and easily repeatable test compared to tissue test, could identified somatic mutation detected on the circulating tumor DNA (ctDNA) extracted from a plasma. This approach should better represent the heterogeneity of the tumor compared to the primary biopsy and maybe helpful in monitoring the onset of potential mutations involved in treatment resistance. Furthermore, ctDNA may inform about timing and potential cooperation of multiple driver genes aberration guiding the treatment options in patients with mCRPC. However, the clinical use of ctDNA test in prostate cancer compared to blood and tissue testing are currently very limited. In this review, we summarize the current therapeutic indications in prostate cancer patients with DDR deficiency, the recommendation for germline and somatic-genomic testing in advanced PC and the advantages of the use liquid biopsy in clinical routine for mCRPC.

## 1 Introduction

Deleterious aberrations in DNA repair genes are found in a considerable rate of patients with advanced prostate cancer (PC) (1–3). With the advent of target therapy such as ribose polymerase poly-ADP inhibitors (PARPis) and immune checkpoint inhibitors (ICIs), genomic testing has become part of the clinical practice in metastatic castration resistant prostate cancer (mCRPC) patients with DNA damage repair (DDR) (4). Homology recombination repair (HRR) is the DDR mechanism most frequently altered in PC and mutation of the *BRCA2* gene is the most frequently detected among the DDR genes (5). Oppositely to *BRCA1* involvement, the germline *BRCA2* mutations have been associated with a 2 to 6 fold increase in the risk for PC (6). *BRCA2* mutant patients seems to have a more aggressive phenotype, and a significant reduction in survival times compared to the non-mutated patients (7–9). Others germline mutations such as ataxia mutated telangiectasia (*ATM*), checkpoint kinase 2 (*CHEK2*), and the partner and locator of *BRCA2* (*PALB2*) seems to correlate, albeit to a lesser extent, with an increase of the risk of PC (3, 10, 11). Currently, the peripheral blood samples are preferentially used to detect germline mutations; while somatic alterations are assessed by extracting DNA from the tumor tissue sample, whose detection could be affected by the sample availability and by tumor heterogeneity.

Liquid biopsy is a new approach increasingly used in clinical setting allowing the rapidly and/or simultaneously detection/capture of cell-free DNA or circulating tumor cells (CTCs) or DNA (ctDNA), and extracellular vesicles. The liquid biopsy is a less invasive molecular profiling resource able to obtain intratumoral heterogeneity and to track dynamic changes and resistance mechanism occurring during therapies (12). The ctDNA has become a viable option to perform genomic testing in PC patients, receiving Food and Drug Administration (FDA) approval in the last years (13). However, although the advantages are now known, several limitations to the use of the ctDNA test are still present.

Here, we review the role that liquid biopsy currently plays in PC, the reliability of the ctDNA test in detecting DDR mutations and the evidence in favor of its routinely introduction in clinical practice.

## 2 DNA damage repair deficiency and mutations in prostate cancer

The DNA repair process is a fundamental mechanism for identifying and correcting the DNA damage induced by environmental factors or normal cellular metabolic processes. DNA damage induces a complex cascade of signals involving various checkpoints capable of interrupting the cell cycle to guarantee the repair of the lesion or, if not possible, to induce senescence and apoptosis (14).

**Figure 1:**
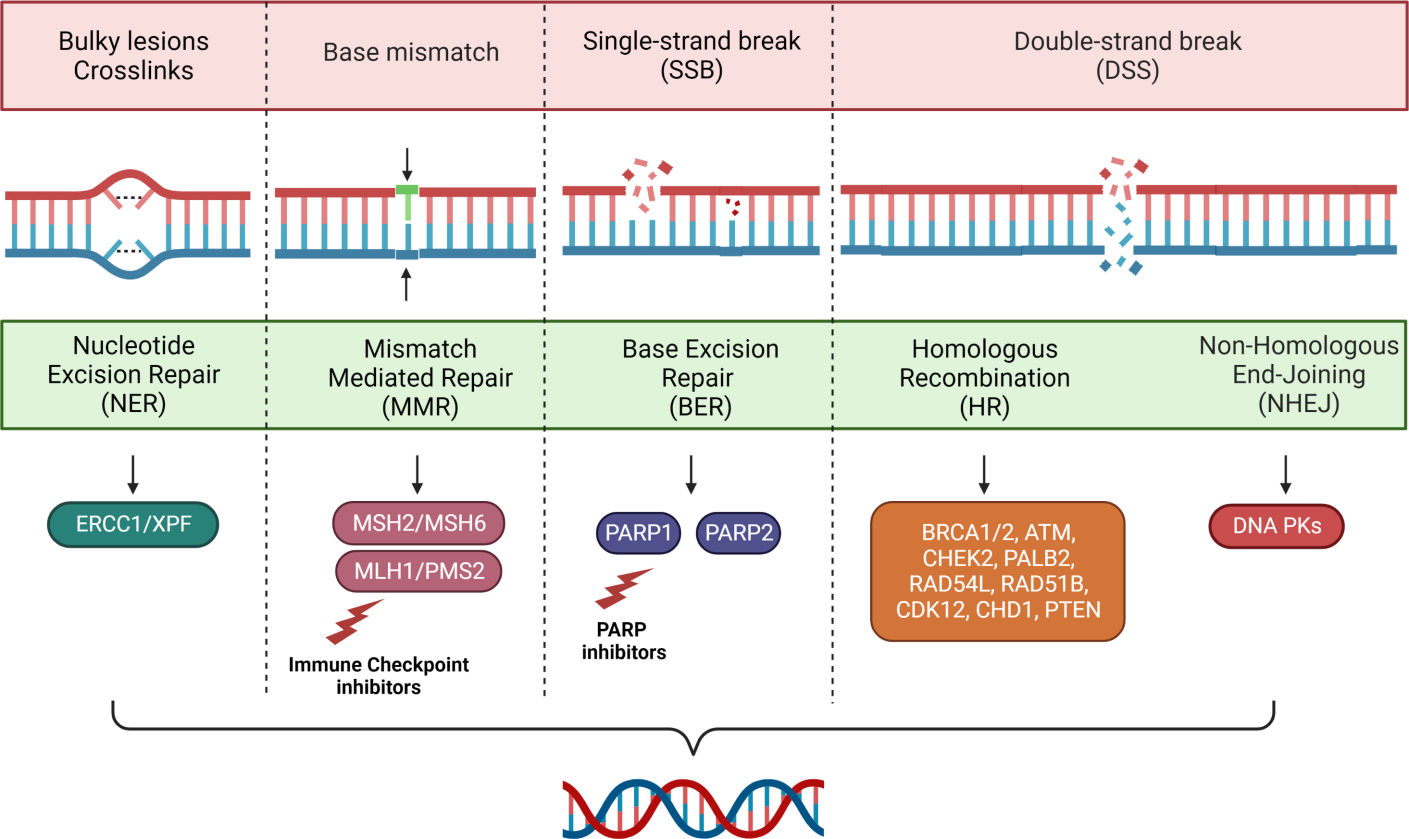
Main mechanisms of DNA repair.

This process is critical for cell survival, as it promotes genomic stability and reduces the risk of inheriting damage during cell division. DNA repair pathways include single-stranded break (SSB) defect repair mechanisms (base excision repair, nucleotide excision repair and mismatch repair (MMR) and repair mechanisms for damage to the double stranded (DSB) (homologous recombination (HR) and non-homologous end joining) as dispatched in **Figure 1**. Other mechanisms such as direct chemical inversion and crosslink repair between strands, although less common, may be coincided in the removal of damage (15). However, the presence of cells with alterations in these pathways are related to fallible repair mechanisms with consequent accumulation of cellular mutations and tumor transformation.

MMR is a proteins system including MLH1, MSH2, MSH6, and PMS2, recognizing and repairing erroneous insertion, deletion, and mis-incorporation of bases caused by DNA polymerase during the DNA replication (16). Its alteration, represented phenotypically as microsatellite instability (MSI), has been firstly identified in tumors from patients with Lynch Syndrome and subsequently in different types of cancers becoming an overall biomarker of response to treatments (17).

In the Memorial Sloan Kettering Cancer Center (MSKCC) cohort, the MSI and defective MMR (dMMR) have been identified in approximately 5% of PC patients (18). Another prospective case series reported MSI-H/dMMR in 3.1% of PC patients, while an unselected cohort study of 3,607 patients with personal history of PC reported the presence of mutations germline in MLH1, MSH2, MSH6 or PMS2 in 1.7% of cases (19, 20). On 60 rapid tumor autopsies from metastatic PC patients, 12% resulted dMMR/MSI-H (21), while in another study on 150 mCRPC patient’s tumor biopsy detected an MSI-H frequency of 3% (22). This high variable frequency of dMMR/MSI-H ranged from 1 to 12% in patients with mCRPC may be in part explained by the diversity of assays used to detect tumors with dMMR (23, 24).

HR is a complex DNA double helix repair system, allowing one stretch of the DNA double helix to serve as a template to restore lost or damaged information in the other stretch. The germinal alterations in various genes belonging to HR, mainly BRCA1 and BRCA2, have been associated with the development of familial tumors, primarily involving breast and ovarian cancer, and subsequently prostate and pancreas cancer (25). Failure in the HR repair system can compromise the elimination of genome mutations, favoring the accumulation of DNA damage events and oncogenesis (26).

Actionable molecular alterations and aberrations in HR and MMR pathways occur in a considerable fraction of localized prostate cancers and, even more frequently in metastatic disease (22).

Regarding the inactivation of HR-associated genes (i.e., BRCA1/2, ATM, CHEK2, PALB2, RAD54L, RAD51B, CHD, CDK12 or PTEN), a number authors have reported the frequencies of somatic and germline mutations at several disease stages of PC (27). Somatic mutations were recorded in 19% of localized PCs and 23% of mCRPC cancers, with the highest incidence in the BRCA2 and ATM genes (22, 23). BRCA2 somatic mutations are associated with germline mutations in 42% of patients with mCRPC (22) and in 60% of localized PC (23). Recent data indicate that 11.8% of patients with metastatic prostate cancer (mPC) have germline mutations in 1 of 16 DNA repair genes: (BRCA1, BRCA2, ATM, CHEK2, PALB2, RAD51D, ATR, and NBN, PMS2, MSH2, MSH6, GEN1, RAD51C, MRE11A, BRIP1, or FAM175A) (28). In the Cancer Genome Atlas (TCGA) cohort, patients with high-risk localized PC had a rate of germline DNA repair mutations of 6% versus 2% in those at low/intermediate risk (23, 28). Another study reported a rate of pathogenic germline mutations in MUTYH, ATM, BRCA1, BRCA2 and BRIP1 of 7.2% in patients with high-risk, very high risk or metastatic PC (29). Other authors have reported varying incidence rates of DDR mutations ranging from 11 to 28%. Robinson et al. found a rate of 22.7% of germline DDR mutations or somatic mutations in BRCA1, BRCA2, ATM, FANCA, CDK12, RAD51B and RAD51C in patients with mCRPC (22), while a rate of less than 11.8% of germline mutations in at least one DDR gene has been reported by Pritchard et al. in the same context of patients (28). In all stages of PC, Abida et al. found germline or somatic alterations in BRCA1/2, TMJ and CHEK2 in 27% of patients (30). More recently in the PROfound study, 28% of mCRPC patients had alterations in 15 genes with direct or indirect roles in HR (31).

## 3 Recommendations for germline and somatic genomic testing in advanced prostate cancer

A family history of prostate cancer, as well as the hereditary breast and ovarian cancer syndrome (HBOC) due to germline mutations in HR genes and Lynch Syndrome increases the risk of PC (32–34). However, approximately 30% of patients with mPC carrying the germline DDR had no family history of cancer. An increased risk of PC has been found in Ashkenazi Jews in whom more than 2% carry germline mutations in *BRCA1* or *BRCA2* and in PC with intraductal histology that appear to have greater genomic instability compared to those with adenocarcinoma histology (35–38). Moreover, a correlation between clinical pathological features (Gleason score ≥8, lymph node and distant metastases to the diagnosis) and germline *BRCA2* mutations has been observed, although the mutation cannot be excluded in the other patients (39). Below reported the main recommendations on carrying out the genetic and/or somatic test (40–42).

### 3.1 Germline testing

National Comprehensive Cancer Network (NCCN) panel recommends germline genetic testing, with or without pretest genetic counseling, for patients with PC and any of the following: a positive family history (multiple family members diagnosed with castration sensitive PC at the age <60 years, a family member died from PC, family history of high-risk germline mutations or of multiple cancers on the same side of the family); high-risk, very-high-risk, regional, or mPC regardless of family history; Ashkenazi Jewish ancestry and intraductal histology. Germline testing should include proteins of MMR and the HR genes (i.e., *BRCA2, BRCA1, ATM, PALB2*, and *CHEK2)*. A cancer predisposition next-generation sequencing (NGS) panel testing, at a minimum including who consider other genes in addition (i.e., *HOXB13)* to the above, and guided by clinical context can be considered (40, 43, 44).

### 3.2 Somatic tumor testing

Alternatively somatic tumor test follows these recommendations: tumor testing for somatic HR gene mutations (i.e., *BRCA1/2, ATM, PALB2, RAD51D*, *FANCA*, and *CHEK2*) and MSI/dMMR can be considered in patients with regional or mPC; multigene molecular testing can be considered for patients with low- and favorable-intermediate risk PC and life expectancy ≥10 years; the Decipher molecular assay can be considered as part of counseling for risk stratification in patients with prostatic specific antigen (PSA) resistance/recurrence after radical prostatectomy. If HR mutations, especially *BRCA1/2, ATM, CHEK2*, or *PALB2* are found, patient should be referred for genetic counseling to assess for the possibility of hereditary tumors such as HBOC. MSI testing should be performed using an NGS assay validated for prostate cancer and if positive, the patient should be referred for genetic counseling to assess for the possibility of Lynch Syndrome (40, 45–47).

Overall, the current recommendations are summarized below:

- Germline testing for DDR genes associated with cancer predisposition syndromes (especially *BRCA2*) is recommended for patients with a family history of cancer and should be considered in all patients withmPC;

- Somatic testing for HR and MSI genes should be considered in all patients with mCRPC;

- Patients with pathogenic mutations detected on somatic testing should be referred for germline testing and genetic counselling;

- In patients with localized prostate cancer, tissue molecular tests can be considered in the presence of suspected clinicopathological factors to aid decision making.

Notably European Association of Urology (EAU) reported genetic testing on circulating tumor DNA (ctDNA) as an alternative to tissue testing although still less common (42).

## 4 Damage of DNA damage repair as therapeutic target in prostate cancer

### 4.1 PARP inhibitors

PARP system is a nucleolar proteins complex involved in DNA repair, genomic stability and programmed cell death (48). Its main role consists in detecting and initiating an immediate cellular response to SSB damage (48). PARP inhibitors have developed as a possible therapeutic strategy in patients with DDR.

The anticancer effect of these drugs is attributed to the catalytic inhibition of PARP that interfere with efficient DNA damage repair inducing tumor cells death (49). While in normal cell PARP inhibition is tolerated, in tumors cells with concomitant HR alteration, the effect of PARPi are notable (50):the defective enzymatic function of PARP results in the accumulation of SSB that promote the accumulation of damage in the potentially lethal DSB, preferentially repaired by HR (51). The concomitant loss of PARP function in cancer cells with altered HR proteins involved in HR deficient repair with the accumulation of DSBs and subsequent cell death (51).

Based on this synergistic effect numerous PARP inhibitors (i.e., olaparib, rucaparib, niraparib, talazoparib and veliparib) have been tested firstly in patients with germline mutations in *BRCA1* or *BRCA2* (52, 53). Afterwards, sensitivity to PARPi has been proved in tumor with loss of other tumor suppressor DNA repair proteins (*e.g.*, *ATR, ATM, RAD51, CHEK1/2, and PALB2*), suggesting the validity of this therapeutic strategy also in patients intrinsically deficient in HR without *BRCA1/2* mutations (54–57). The outcome benefits observed with PARPi in DDR mutated breast and ovarian cancers, led to the evaluation of PARPi efficacy in PC.

TOPARP-A was the first phase II study to test in 2014 olaparib in patients with mCRPC regardless of DDR mutations. Fourteen of the 16 patients with aberrations of the DNA repair genes (*BRCA2, ATM, BRCA1 or CHEK2 and HDAC2*) achieved a response to treatment measured by a composite methodology including the decline of CTCs (58). Based on these findings, olaparib received the FDA’s breakthrough PC therapy designation (59).

A subsequent phase II study, TOPARP-B, further examined the anticancer effects of olaparib in mCRPC patients with DDR mutations who had progressed to an earlier line of therapy (60). Patients positive for pathogen mutation or homozygous deletion in a DDR gene tested with NGS on primary tumor biopsies received olaparib 300 or 400 mg twice daily. Subgroup analysis showed that patients with the *BRCA1/2* mutation predicted greater responses and a longer median radiographic progression-free survival (PFS), with an overall response rate (ORR) of 83.3%. In patients with *ATM* and *PALB2* mutations, rate of radiographic objective responses was 8.3% and 33,3%, respectively; while PSA declines of at least 50% were detected in 5.2 and 66.6% of patients with alteration of *ATM* and *PALB2*, respectively, suggestinga susceptibility of *PALB2*to PARP inhibition. Limitations in obtaining accurate and timely somatic genetic testing in this trial allowed to enlist only 13.7% (98/711) of the screened patients. Data derived from these trials showed the antitumor effects of olaparib both when used to treat mCRPC patients with certain DDR genetic aberrations and in some patients with non‐BRCA mutations (60).

The phase III study, PROfound, tested the efficacy of olaparib versus androgen receptor signaling inhibitors (ARSi) (abiraterone or enzalutamide) in patients with mCRPC and mutations in 15 HR-associated genes (*BRCA1, BRCA2, ATM, BRIP1, BARD1, CDK12, CHEK1, CHEK2, FANCL, PALB2, PPP2R2A, RAD51B, RAD51C, RAD51D* and *RAD54L*) (31). The primary endpoint examined was imaging-based PFS. The patients were divided into two cohorts: the cohort A included patients with alterations in *BRCA1/2* or *ATM*: the cohort B included patients with alterations in any of the other 12 genes. All patients received 300 mg olaparib twice daily versus second ARSi in a 2:1 ratio. In the overall population (cohorts A and B), significantly longer PFS was recorded in patients treated with olaparib compared to control arm (5.8 vs 3.5 months; hazard ratio [HR], 0.49; 95% confidence interval [CI], 0.38-0.63; p<0.001). An even longer PFS was recorded in cohort A in the olaparib group compared to control (7.4 vs 3.6 months; HR, 0.34; 95% CI, 0, 25-0.47; p <0.001) as well as a better OS (18.5 vs 15.1 months; HR, 0.64; 95% CI, 0.43 to 0.97; p=0.02). Notably, failure to sequence DNA occurred in approximately 31% of the tumor samples. Based on these results, FDA recently approved olaparib for patients with mCRPC progressed to enzalutamide and/or abiraterone who have deleterious germline alterations in *BRCA1*/*2* or somatic deleterious alteration in *BRCA1/2, ATM, BARD, BRIP, CDK12, CHEK1, CHEK2, FANCL, PALB2, RAD51B, RAD51C, RAD51D*, and *RAD54L* (61).

Another PARP inhibitor, rucaparib, has been evaluated for the treatment of patients with mCRPC who have germline or somatic mutations in the DDR genes (62). Phase II study, TRITON2 enlisted patients with any mutation in the HR genes, showing initial efficacy and safety results that allowed for the designation of rucaparib as a breakthrough therapy by the FDA. Preliminary data from this study showed promising results: 43.9% of patients with *BRCA* achieved a confirmed radiographic response, and lasting responses (62). Partial radiographic responses have been observed in 10.5% of patients with non-*BRCA* DDR genes and patients with *ATM* mutations. Two patients with *CHEK2* aberrations had a confirmed partial response and a confirmed PSA response. No objective response was observed in patients with *CDK12* mutations. In the 13 patients whit other mutations including *FANCA, PALB2, BRIP1*, or *RAD51B*, ORR was 38.5% with one complete response and four partial responses. The FDA recently approved rucaparib for the treatment of mCRPC patients with germline or somatic *BRCA1/2* mutations (63). Is ongoing a phase III study, TRITON3, comparing rucaparib with standard of care treatments, enrolling only patients with mCRPC and mutations in *BRCA1/2* and *ATM* (NCT02975934).

The GALAHAD study evaluated the activity of niraparib in patients with mCRPC and DDR gene alterations received three or more systemic therapies for mCRPC (64). In this phase II trial niraparib was tolerable and showed anti-tumour activity in heavily pretreated patients, particularly in those with *BRCA* alterations with an ORR of 34.2%. Niraparib combined with abiraterone acetate/prednisone versus abiraterone acetate/prednisone for patients with mCSPC and deleterious germline or somatic HRR gene mutated is under evaluation in the phase III trial, AMPLITUDE (NCT04497844).

Finally, talazoparib has been tested in TALAPRO-1 phase II study enrolling patients with measurable soft tissue disease, progressive mCRPC, and DDR mutated (*ATM, ATR, BRCA1/2, CHEK2, FANCA, MLH1, MRE11A, NBN, PALB2, RAD51C*), treated with one or more taxane-based chemotherapy regimens and ARSi for mCRPC to receive oral talazoparib 1 mg/day until radiographic progression, unacceptable toxicity, consent withdrawal, or death (65). Talazoparib monotherapy has encouraging antitumor activity in docetaxel-pretreated mCRPC patients with *BRCA1/2* alterations and was generally well tolerated. The efficacy and safety of talazoparib and enzalutamide combination in mCRPC patients with or without DDR mutations is currently under evaluation in the phase III trial TALAPRO-2 (NCT03395197). The phase III study, TALAPRO-3, is comparing talazoparib plus enzalutamide versus placebo plus enzalutamide in patients with mCSPC and DDR alterations (NCT04821622).

As the use of PARPi is limited by primary resistance mechanisms and the onset of secondary resistance in sensitive patients’numerous efforts have been aimed at developing combined treatment approaches (66)

PROpel is a phase III trial randomizing patients with mCRPC regardless of HRR status to receive olaparib or placebo and abiraterone plus prednisone or prednisolone (67). The primary endpoint was investigator-assessed radiographical (rPFS), OS was one of the multiple secondary endpoints. Treatment with olaparib plus abiraterone significantly prolonged rPFS in patients with mCRPC regardless HRR status compared to control (24.8 vs 16.6 months; HR, 0.66, 95% CI, 0.54-0.81; p <0.0001). The safety and tolerability profile of combination was consistent with the known safety profiles of the individual drugs.

MAGNITUDE is a randomized, double-blind phase III study enrolling mCRPC patients (≤4 months of prior abiraterone acetate/prednisone for mCRPC was allowed) with or without HRR biomarker positive (*ATM*, *BRCA1/2, BRIP1, CDK12, CHEK2, FANCA, HDAC2, PALB2*) to receive niraparib 200 mg once daily plus abiraterone acetate/prednisone or placebo plus abiraterone acetate/prednisone (68). The primary endpoint was rPFS. assessed by blinded independent central review in the *BRCA1/2* group then in all patients with positive HRR biomarkers. The preplanned futility analysis in HRR mutations negative patients showed no benefit of adding niraparib to abiraterone acetate/prednisone in the prespecified composite endpoint (PSA progression or rPFS: HR 1.09, 95% CI 0.75-1.59). Niraparib plus abiraterone acetate/prednisone significantly improved the primary clinical outcome in HRR biomarker positive patients, with a manageable safety profile and health-related quality of life. Therefore, while PROpel trial showed a global benefit of PARPi and ARSi without the need for HRR stratification in untreated mCRPC patient, the MAGNITUDE study results support the combination strategy only for patients with alterations in HRR genes.

### 4.2 Immune checkpoint inhibitors

Prostate cancer belong to those tumors whose microenvironment is defined as immune-excluded, as it is characterized by a low mutational load, a reduced expression of neoantigens, hyperactivity of myeloid-derived suppressor cells and T-regulator cells, loss of major histocompatibility complex class I expression and abnormal IFN-1 signaling (69). However, like other solid tumors, it has been shown that dMMR or MSI-H prostate cancer may respond better to the immune checkpoint blockade (70).

Based on the results of a phase II trial, KEYNOTE 158, pembrolizumab received the first tissue agnostic approval for an antineoplastic therapy granted by the Food and Drug Administration (71). Patients enrolled in this study presented several types of cancer, including PC in two cases, with MMRd and had received at least one prior therapy. Objective radiographic responses have been reached in 46 (53%) of patients, with 18 (21%) achieving a complete response. Two subsequent placebo-controlled, phase III trials testing CTLA-4 inhibitor, ipilimumab, did not find improvement in OS in mCRPC patients, while pembrolizumab as single-agent showed a low response rate of 3%-5% post chemotherapy (72–74). A phase II trial combined ipilimumab with nivolumab showed an overall response of 26% in mCRPC chemotherapy naïve, although with an significant rate of grade ≥3 adverse events (75). Follow-up studies have largely confirmed pembrolizumab’s efficacy in men with MMRd prostate cancer. In a study conducted at MSKCC, 3.1% of enrolled mCRPC patients were characterized by MSI-H PC and 11 of these received ICI-based therapy (19). About half of the patients achieved a PSA decline of at least 50% from baseline (PSA50 response), and four patients achieved a radiological response. A small sample size-based study by Schweizer et al. showed that 4 out of 10 ductal PC patients were dMMR, and 3 of them were also characterized by MSI-H. Notably, one of these dMMR/MSI-H patients with ductal PC achieved a significant reduction in PSA levels during treatment with pembrolizumab (76). Several other series of studies enrolling patients with PC with MMRd reported a PSA50 response, ranging from 50 to 65% when treated with ICI monotherapy, with long term responses observed (77).

## 5 DNA damage repair deficiency testing type

### 5.1 Tissue and whole blood testing

Tests for identifying mutations in DNA repair genes can be detected on two main tissues: blood or tumor tissue. The main difference between these two strategies is based on the type of mutation that can be detected. Through blood analysis we can detect genomic rearrangements significant for patients and their family, without indications on somatic mutations. Both alterations can be identified through tissue testing (78). The blood test has numerous advantages including the easy availability of the sample, the minimum invasiveness of the procedure and the repeatability of the test. Tissue testing can be performed on both surgical or biopsy specimens of the prostate and on metastatic sites, although with some limitations. Firstly, the multifocal nature of PC, which may result in the core biopsy analyzed not representing the metastatic disease clone (79). The execution of the biopsy on the metastatic site is an invasive procedure and not free from complications for the patient, in addition the frequent bone involvement as a metastatic site in patients with PC considerably reduces the probability of success of the test (78). Secondly, is related to quantity and quality of sample. In fact, the small size of prostate primary tumor biopsies and progressive degradation of DNA in paraffine after years, are two conditions to be carefully considered for the choice of the test. Contrary to the high quality of blood sample, quality in the tissue is low and variable in relation to factors including carrying out different molecular tests, presence of necrosis, high infiltration of inflammatory cells, degradation related to aging, poor formalin fixation and cauterized tissue (80, 81). This making up to 20-30% of core biopsies unsuitable for NGS testing using commercial platforms (31).

Regarding MSI the gold standard for determining MSI/dMMR status is immunohistochemistry (IHC) and polymerase chain reaction (PCR) testing performed on tumor tissue samples. IHC is highly sensitive and specific in Lynch Sindrome-associated tumors by exploring the expression of the four major MMR proteins or just the MSH6/PMS2 doublet (82, 83). The MSI-PCR method based on PCR amplification of microsatellite regions followed by capillary electrophoresis is a reliable alternative to IHC. PCR helps also to recover cases that can escape IHC due to preanalytical problems, indeterminate results, as well as false negatives (non-truncating missense mutations in MMR genes associated with intact antigenicity) (84). Recently, they have emerged new molecular approaches (histopathology-based approach, PCR-based test, NGS-based approaches computational tools for MSI diagnosis) on tumor tissue samples that could improve sensitivity and specificity compared to conventional tests, representing a valid future option.

To date, circulating free DNA (cfDNA) can also be used to accurately determine MMRd and MSI status with the advantage of being easily obtainable compared to a metastatic biopsy; however, there are technical limitations of ctDNA-based sequencing approaches such as the low tumor burden which can results in indeterminate results.

### 5.2 Liquid biopsy and ctDNA

In the last year liquid biopsy has emerged as a promising surrogate for tumor biopsy, capable of overcoming spatial and temporal heterogeneity by allowing longitudinal monitoring of the disease through iterative sampling (85–87). It is an emerging field in the management of patients with cancer and its relevance as a potential diagnostic, prognostic, monitoring, and therapeutic tool makes it an attractive strategy in the management of these patients (87–89). However, liquid biopsy still has some limitations, although seem to be within the reach of technological development soon. This strategy has shown to be able to reliably represent the tumor microenvironment and its modification. Different biomarkers such as CTCs, extracellular vesicles, ctDNA, circulating tumor RNA can be analyzed through liquid biopsy on the blood or on the other human fluids (*i.e.*, urine, sperm, etc.) for diagnostic, prognostic, and predictive purposes (88).

The detection of cell-free DNA (cfDNA) as a part of liquid biopsy in PC has been widely explored, despite its diagnostic value for PC remains controversial. cfDNA is the total amount of circulating DNA found in blood plasma representing the total DNA released by normal and cancer cells. Its concentration may be increased in stroke, trauma, myocardial infarction, and autoimmune diseases (90–92), and even more in patients with advanced cancer (93, 94). Circulating tumor DNA is plasmatic DNA derived specifically from the primary or secondary site of the tumor, or from circulating tumor cells. It can represent 0.01% up to 90% of total free DNA, with an inherent patient variability due to various factors such as location, size, vascularity, tumor stage and response to therapy. The ctDNA level is higher in metastatic cancer than in localized disease and appears to correlate with disease progression (95–98). The release of free DNA from circulating cells can occur passively, during apoptosis or necrosis, or through active secretion. The DNA fragments released during apoptosis differ from those poured into the circulation in case of necrosis in the shorter length (99). A smaller amount of ctDNA is released through active secretion from extracellular vesicles, such as exosomes and prostasomes (100).

Liquid biopsy and specifically ctDNA testing can ensure monitoring of tumor evolution during therapy bypassing the intratumoral heterogeneity that limits tissue testing, especially if performed on the primary site. This allows to ctDNA to also detect resistance mutations. Both somatic and germline mutations can be detected through the ctDNA test, considering the pros and cons of the test (**Table 1**). Among the advantages of this method, there are: readiness in obtaining samples, repeatability during therapy or disease progression and rapidity, 1-2 weeks for the ctDNA test compared to 2-4 weeks for the examinations of the blood (101–103). The main disadvantages are related to amount of DNA and ctDNA levels (104). The amount of DNA in ctDNA is usually a very small fraction of cell-free DNA, especially in the early stages of the disease (105). The level of ctDNA is critical for performing the test, indeed it may vary during treatment and appears to closely correlate with tumor response (106). Some authors have shown how ctDNA determination changes at various stages of treatment. CtDNA was detected in 74% of patients before anti androgen therapy (ADT) initiation versus 59% of patients who received ADT prior to collection, with significantly higher ctDNA fraction in treatment-naïve patients (1.0% vs 11%; p = 0.02). The reduction in the ctDNA fraction was more pronounced after one week of ADT (107, 108).

**Table 1:** Pros and cons of tissue, blood, and ctDNA-based HHR gene tests.

	Tissue	Blood	ctDNA
Mutation detected	Somatic and germline	Germline	Somatic and germline
Sample quantity	Medium	High	Low
Sample quality	Low	High	Variable
Time to response	2-8 weeks	2-4 weeks	1-2 weeks
Advantages	Archivial tissue for tumor histology	Easy to obtain samples Feasiblility in all cases Minimally invasive Easly repeatable	Easly to onbtain samples Better representative o ftumor heterogeneity and metastatic sites Minimally invasive Easly repeatable
Limitations	Tumor heterogeneity Invasive procedute to obtain samples High percentage of tests failed	Does not detect HRRm of somatic origin Does not capture the potential changing genetic profile of disease progression	Low concentration of ctDNA Type of sentitive tests False positive Adeguate amount of ctDNA particularly in early stages.
Genetic counselling	After germline test confirmation	Required	After germline test confirmation

ctDNA, circulating tumor DNA; HRR, homologous recombination repair.

Another critical point lies in the interpretation of the test result. Indeed, a negative result does not exclude the presence of a mutation in the patient’s tumor, while a positive result for gene alterations does not distinguish between germinal and somatic origin. In the former case the patient should receive a confirmatory tissue test, in the latter case they should be referred to an appropriate confirmatory test if a germline mutation is suspected.

Some authors have evaluated the false positive rate linked to specific ctDNA tests in healthy controls, recording a rate of 0.82% in unique short variants. These false positive results may derive from somatic non-tumor changes in genes derived from clonal hematopoiesis indeterminate potential (CHIP), including *ASXL1, ATM, CBL, CHEK2, DNMT3A, JAK2, KMT2D, MLL2, MPL, MYD88, SF3B1, TET2, TP53 and U2AF1* (109–113). Despite tumor biopsy represents the reference tissue for the determination of MSI, clinically it has several limitations mainly related to the complexity of the procedure and to the spatial heterogeneity of the disease (114). Furthermore, in some rare cases, sporadic tumors may have a late onset of MMR defects that tissue biopsy cannot detect, leading to a misclassification of the MSI. It is now known the clinical potentiality of liquid biopsy in establishing tumour molecular diagnostics albeits data regarding its utility in determining MSI status which are still unclear, especially in the prostate cancer.

## 6 Circulating tumor DNA in prostate cancer

In PC, tissue testing is currently the test of choice for the analysis of tumor genomic profiles, although several critical issues have emerged in the main studies. In fact, in 30% of PC cases in which the tissue test was performed before enrollment, it failed due to problems in the pathological review, and during and after DNA extraction (31, 115–117). Therefore, the possibility of evaluating molecular alterations using ctDNA has made its way among pathologists and clinicians (118). NGS of ctDNA from plasma provides a minimally invasive method to identify genomic profile and resistance mechanisms in patients with mCRPC (119). However, the fraction of ctDNA in mCRPC patients and the clinical validity of the genomic alterations detected in plasma remain still unclear.

Several authors have studied the level of agreement between the plasma and tissue testing (**Table 2**). Firstly, Wyatt et al. in their study reported a high concordance rate between the ctDNA test and the metastatic tissue test. A ctDNA rate greater than 2% of the cfDNA was present in 75.6% of the samples (123). In these patients, all somatic mutations identified in metastatic tissue biopsies were simultaneously present in the ctDNA. The concordance results stratified by variant, showed a high positive agreement for substitutions (92%) and indels (95%) and a much lower agreement for rearrangements and copy number loss. Negative concordance was of 100%. In several patients, ctDNA sequencing revealed robust changes do not present in solid biopsy including clinically relevant alterations in the AR, WNT and PI3K pathways (123). Similarly, Vandekerkhove et al., reported a rate of 80% of concordance for mutation detection in diagnostic prostate tissue and ctDNA (107).

**Table 2:** Summary of the studies evaluating concordance between tissue and cDNA testing.

Study (ref)	Patients	Number of samples	Types of tests	Method	Results
Wyatt et al., 2017 **(** 118 **)**	mCRPC	45	Metastatic tissue and ctDNA	Targeted sequencing across 72 clinically relevant genes	All the somatic mutation identified in matched metastatic tissue biopsies were concurrently present in ctDNA; concordance of 88.9% for individual gene CAN.
Vandekerkhove et al., 2019 **(** 119 **)**	mCSPC	53	Diagnostic prostate tissue and ctDNA	Targeted sequencing strategy capturing the exon of 73 driver genes	80% of concordance for mutation detection in the matched samples. Combined ctDNA and tissue analysis identified potential driver alterations in 94% of patients; ctDNA or prostate biopsy alone failed in the 36% of cases.
Tukakinski et al., 2021 **(** 120 **)**	mCRPC	3334 (1674 screening samples from TRITON2/3 trial)	Tissue biopsy and ctDNA	Plasma assay with 62 (FoundationACT) or 70 genes (FoundationOne Liquid)	93% of concordance between BRCA 1/2 mutations detected in tissue biopsy and those identified by ctDNA 100% of concordance for germline variants. In 20 patients, BRCA 1/2 gene alterations were identified using ctDNA but not tissue testing.
Schweizer et al., 2021 **(** 121 **)**	mCRPC	51	Primary prostate tissue, metastatic tissue and ctDNA	Plasma assay with 324 (FoundationOne CDx) or 70 genes (FoundationOne Liquid)	Of the 53 paired samples, at least partial concordance in DDR genes was identified in 43 cases (84%) Concordance was numerically higher between ctDNA primary pairs compared with metastatic primary pairs; however, this difference was not statistic ally significant (92% vs 79%. 2 monoallelic DDR gene alterations only found in primary tissue.
Warner et al., 2021 **(** 122 **)**	mCRPC	1615	Archival primary tissue and ctDNA	Plasma assay with 22 genes (Illumina MiSeq or HiSeq 1500/2500 machine)	DDR gene status was concordant (94%) between archival primary tissue taken at cancer diagnosis and serial ctDNA-positive samples collected in the mCRPC setting.

mCSPC, metastatic castration sensitive PC; mCRPC, metastatic castration resistant prostate cancer; ctDNA, circulating tumor DNA; DDR, damage DNA repair.

De Bono et al. reported a very high agreement between tissue and ctDNA testing for the detection of deleterious alterations in *BRCA1* or *BRCA2* with a positive percentage agreement of 88% and a negative percentage agreement of 95%. Some degree of discrepancy has been attributed to biological differences and sampling times between tumor tissues and plasma samples (31). Likely, Mateo et al. reported a similar prevalence between NGS over 470 primary tumors and metastatic site biopsy findings in patients who later developed mCRPC (60).

In their large study of ctDNA in 3334 mCRPC patients Tukakinsky et al. showed a high agreement between the alterations identified by liquid biopsy and those detected by tissue biopsy (119). The 94% of patient plasma samples had detectable ctDNA. In 79.5% of all patients, liquid biopsy identified at least one genomic alteration (*Tp53, RA, BRCA2/1, PI3K/AKT/mTOR, WNT/β-catenin pathway genes, RAS/RAF/MEK, MSI-H*). Regarding BRCA mutations, 67 (8%) *BRCA1/2* alterations were detected in both tissue and liquid biopsy, 5 (0.6%) exclusively in tissue biopsy (in 4 samples the ctDNA fraction was less than 1%) and 20 (2.4%) exclusively in liquid biopsy. The 20 cases detected only with liquid biopsy, may represent secondary alterations to the collection of the tissue sample. The concordance between BRCA mutation identified by blood test and ctDNA analysis was 100%.

Warner et al. demonstrated that the frequency of harmful *BRCA2, ATM* and *CDK12* changes detectable in plasma ctDNA was like those observed in the population with metastatic tissue biopsy in a large cohort of mCRPC, supporting minimally invasive liquid biopsy as approaches to identify responders to PARP inhibitors (120).

Finally, Schweizer at al. in their study, showed as primary prostate tissue accurately reflected the mutational status of activatable DDR genes in metastatic tissue. After excluding probable CHIP events, the ctDNA profile accurately detected DDR mutations including alterations suggesting potential related mechanisms of resistance (122). Only one patient developed a *BRCA2* alteration later, while two cases *BRCA1/2* mutation positive in the primary sample, got lost in downstream samples. This may be reconducted to the intraprostatic genomic heterogeneity. However, it is also plausible a selective therapeutic pressure in the first case and an eradication of clones sensitive to DNA-damaging therapies in the second.

The confirmation of the high prevalence of HRR-associated gene mutations in advanced PC has led to some controversy regarding the use of archival primary prostate tumor biopsies for genomic profiling once patients have developed mCRPC (30, 121, 124).

Recently, Hussain et al. tried to outline the correct use of tissue for mutational analyzes by formulating the following recommendations (125): -in presence of more samples with similar tumor content, the choice must fall on the younger sample; -if the samples available exceed 5 years from collection, it is necessary to use those with the highest tumor content and high yield of DNA such as lymph nodes; -for the samples just collected, the recommendation is to optimize the fixation and storage of formalin and avoid descaling.

Regarding MSI, a good overall agreement was observed between conventional tissue-based tests and newly developed ctDNA-based approaches (126–128). This suggested that ctDNA-based MSI diagnosis could be performed as part of clinical practice to identify patients who might benefit from immune checkpoint inhibitors when tissue samples are unavailable or scarce (126, 127, 129). In Nakamura et al., changes in basal MSI levels during ICI treatment has been correlated well with those of other ctDNA markers and reliably reflected tumor response to treatment (128). Longitudinal analysis of ctDNA also allowed to detect the acquisition of somatic MSI that can appear during cancer evolution in patients initially diagnosed with MSS tumors (130). At the present state of knowledge, there are few cases in which the MSI phenotype is acquired during the disease (19, 130). This phenomenon could be partly explained by the fact that most cancers are screened for MSI only at the time of diagnosis, underestimating cases. Further studies are needed to evaluate the true impact of such an acquired MSI phenotype in clinical practice.

In addition to a predictive value, a prognostic value may be recognized to ctDNA. Already in localized disease, *BRCA1/2* germline changes have been associated with poor outcome, including disease progression among patients under active surveillance (131) or a higher risk of recurrence and death among patients undergoing salvage therapy (132). In a retrospective study of mCRPC profiled patients with a 70-gene NGS cfDNA panel an alteration was recorded in over 94% of cases, and a greater number of ctDNA alterations were associated with a shorter time to treatment failure with chemotherapy (HR, 1.05, p=0.026) or androgen inhibitors. In the study conducted by the detection of a ctDNA fraction greater than 30% was strongly associated with a poor response to enzalutamide or abiraterone therapy even after adjustment for other clinical prognostic factors (133). In this study, a ≥50% reduction in cfDNA concentration after eight weeks of therapy was independently associated with longer OS suggesting free DNA concentration as predictive factor to PARPi response.

## 7 Conclusion and future perspectives

Given the significant percentage of mCRPC patients with DNA repair genes mutations and the therapeutic possibilities currently available, the most important guidelines recommend the performance of genomic testing in all these patients. The test can be performed on various samples mainly whole blood and tissue, with relative advantages and limitations. In recent years, ctDNA has become a viable option for performing genomic testing receiving FDA approval in 2020 (13). The ctDNA can overcome the limitations of tissue testing, which can fail in up to a third of cases. Additionally, ctDNA testing can be performed longitudinally by detecting new alterations and resistance mutations that may emerge during disease progression.

Several authors demonstrated high agreement between tissue testing and ctDNA testing suggesting that the analysis on ctDNA is sufficient to identify all DNA alterations and be used as a guide for patient management with mCRPC. Ideally, the combined use of the two techniques could ensure the study of the molecular subtype, paving the way for the implementation of precision therapy, but still far from possible clinical practice.

To consider the ctDNA test results as reliable as possible, it should be performed in certified institutions using the standard NGS procedure. New sequencing technologies such as PacBioScience and Oxford Nanopore allow for the acquisition of additional information, such as large intermediate chromosomal aberrations that appear to correlate with a worse prognosis of PC (134, 135). These new technologies, still burdened by a high sequencing error rate and high costs, will enable the generation of more complete and easy-to-read data.

The large proportion of patients with a rich genomic signal from ctDNA and the sensitive and specific detection of *BRCA1/2* alterations position liquid biopsy as a compelling clinical complement for comprehensive tissue genomic profiling for mCRPC patients. However, despite the findings, there are still several barriers limiting the clinical implementation of genomic sequencing, including cost, access, and feasibility based on often limited tissue availability or quality. Furthermore, only a fraction of patients with PC and genomic aberrations respond durably to targeted therapy.

The integration of analyzes that combine genomics with transcriptome, epigenome and tumor microenvironment study could help identify patients who have more likely to benefit from targeted therapies. In the future, these integrated systems, combined with clinical information, will ensure a further push towards precision oncology.

## Author contributions

GR had full access to all the data in the study and takes responsibility for the integrity of the data and the accuracy of the data analysis. Study concept and design: GR, DG. Acquisition of data: GR, MC, MG, BR, LP. Analysis and interpretation of data: GR, Drafting of the manuscript: GR, MC Critical revision of the manuscript for important intellectual content: DG Statistical analysis: None Obtaining funding: None. Administrative, technical, or material support: None. Supervision: DG, GN. Financial disclosures: None. All authors contributed to the article and approved the submitted version.

## References

[1] Ghose A Moschetta M Pappas-Gogos G Sheriff M Boussios S . Genetic aberrations of dna repair pathways in prostate cancer: Translation to the clinic. Int J Mol Sci (2021) 22(18):9783. doi: 10.3390/ijms22189783 34575947 PMC8471942

[2] Boussios S Rassy E Shah S Ioannidou E Sheriff M Pavlidis N . Aberrations of DNA repair pathways in prostate cancer: A cornerstone of precision oncology. Expert Opin Ther Targets (2021) 25:329–33. doi: 10.1080/14728222.2021.1951226 34225539

[3] Burdak-Rothkamm S Mansour WY Rothkamm K . DNA Damage repair deficiency in prostate cancer. Trends Cancer (2020) 6:974–84. doi: 10.1016/j.trecan.2020.05.011 32517958

[4] Merseburger AS Waldron N Ribal MJ Heidenreich A Perner S Fizazi K . Genomic testing in patients with metastatic castration-resistant prostate cancer: A pragmatic guide for clinicians. Eur Urol (2021) 79:519–29. doi: 10.1016/j.eururo.2020.12.039 33494937

[5] Schiewer MJ Knudsen KE . DNA Damage response in prostate cancer. Cold Spring Harbor Perspect Med (2019) 9(1):a030486. doi: 10.1101/cshperspect.a030486 PMC6314076 29530944

[6] McNevin CS Cadoo K Baird AM Murchan P Sheils O McDermott R . Pathogenic brca variants as biomarkers for risk in prostate cancer. Cancers (2021) 13(22):5697. doi: 10.3390/cancers13225697 34830851 PMC8616097

[7] Petrucelli N Daly MB Pal T . BRCA1- and BRCA2-associated hereditary breast and ovarian cancer. Univ Washington Seattle (1993).

[8] Ibrahim M Yadav S Ogunleye F Zakalik D . Male BRCA mutation carriers: Clinical characteristics and cancer spectrum. BMC Cancer (2018) 18(1):179. doi: 10.1186/s12885-018-4098-y 29433453 PMC5809938

[9] Gallagher DJ Gaudet MM Pal P Kirchhoff T Balistreri L Vora K . Germline BRCA mutations denote a clinicopathologic subset of prostate cancer. Clin Cancer Res (2010) 16:2115–21. doi: 10.1158/1078-0432.CCR-09-2871 PMC3713614 20215531

[10] Cortesi L Piombino C Toss A . Germline mutations in other homologous recombination repair-related genes than brca1/2: Predictive or prognostic factors? J Personalized Med (2021) 11:245. doi: 10.3390/jpm11040245 PMC8066561 33800556

[11] Nombela P Lozano R Aytes A Mateo J Olmos D Castro E . BRCA2 and other DDR genes in prostate cancer. Cancers (2019) 11(3):352. doi: 10.3390/cancers11030352 30871108 PMC6468860

[12] Lone SN Nisar S Masoodi T Singh M Rizwan A Hashem S . Liquid biopsy: A step closer to transform diagnosis, prognosis and future of cancer treatments. Mol Cancer (2022) 21:1–22. doi: 10.1186/s12943-022-01543-7 35303879 PMC8932066

[13] FDA Expands approval of cancer liquid biopsy - NCI (2022). Available at: https://www.cancer.gov/news-events/cancer-currents-blog/2020/fda-foundation-one-cancer-liquid-biopsy-expanded-approval.

[14] Li Z Pearlman AH Hsieh P . DNA Mismatch repair and the DNA damage response. DNA Repair (2016) 38:94–101. doi: 10.1016/j.dnarep.2015.11.019 26704428 PMC4740233

[15] Friedberg EC . DNA Damage and repair. Nature (2003) 421:436–40. doi: 10.1038/nature01408 12540918

[16] Pećina-Šlaus N Kafka A Salamon I Bukovac A . Mismatch repair pathway, genome stability and cancer. Front Mol Biosci (2020) 7:122. doi: 10.3389/fmolb.2020.00122 32671096 PMC7332687

[17] Bonneville R Krook MA Kautto EA Miya J Wing MR Chen H-Z . Landscape of microsatellite instability across 39 cancer types. JCO Precis Oncol (2017) 2017:1–15. doi: 10.1200/po.17.00073 PMC5972025 29850653

[18] Latham A Srinivasan P Kemel Y Shia J Bandlamudi C Mandelker D . Microsatellite instability is associated with the presence of lynch syndrome pan-cancer. J Clin Oncol (2019) 37:286–95. doi: 10.1200/JCO.18.00283 PMC6553803 30376427

[19] Abida W Cheng ML Armenia J Middha S Autio KA Vargas HA . Analysis of the prevalence of microsatellite instability in prostate cancer and response to immune checkpoint blockade. JAMA Oncol (2019) 5:471–8. doi: 10.1001/jamaoncol.2018.5801 PMC6459218 30589920

[20] Nicolosi P Ledet E Yang S Michalski S Freschi B O’Leary E . Prevalence of germline variants in prostate cancer and implications for current genetic testing guidelines. JAMA Oncol (2019) 5(4):523–8. doi: 10.1001/jamaoncol.2018.6760 PMC6459112 30730552

[21] Pritchard CC Morrissey C Kumar A Zhang X Smith C Coleman I . Complex MSH2 and MSH6 mutations in hypermutated microsatellite unstable advanced prostate cancer. Nat Commun (2014) 5:4988. doi: 10.1038/ncomms5988 25255306 PMC4176888

[22] Robinson D Van Allen EM Wu YM Schultz N Lonigro RJ Mosquera JM . Integrative clinical genomics of advanced prostate cancer. Cell (2015) 161:1215–28. doi: 10.1016/j.cell.2015.05.001 PMC4484602 26000489

[23] Abeshouse A Ahn J Akbani R Ally A Amin S Andry CD . The molecular taxonomy of primary prostate cancer. Cell (2015) 163:1011–25. doi: 10.1016/j.cell.2015.10.025 PMC4695400 26544944

[24] Rodrigues DN Rescigno P Liu D Yuan W Carreira S Lambros MB . Immunogenomic analyses associate immunological alterations with mismatch repair defects in prostate cancer. J Clin Invest (2018) 128:4441–53. doi: 10.1172/JCI121924 PMC6159966 30179225

[25] Petrucelli N Daly MB Pal T . BRCA1- and BRCA2-associated hereditary breast and ovarian cancer. Univ Washington Seattle (1993).

[26] Wright WD Shah SS Heyer WD . Homologous recombination and the repair of DNA double-strand breaks. J Biol Chem (2018) 293:10524–35. doi: 10.1074/jbc.TM118.000372 PMC6036207 29599286

[27] Lang SH Swift SL White H Misso K Kleijnen J Quek RGW . A systematic review of the prevalence of DNA damage response gene mutations in prostate cancer. Int J Oncol (2019) 55:597–616. doi: 10.3892/ijo.2019.4842 31322208 PMC6685596

[28] Pritchard CC Mateo J Walsh MF De Sarkar N Abida W Beltran H . Inherited DNA-repair gene mutations in men with metastatic prostate cancer. New Engl J Med (2016) 375:443–53. doi: 10.1056/nejmoa1603144 PMC4986616 27433846

[29] Giri VN Obeid E Gross L Bealin L Hyatt C Hegarty SE . Inherited mutations in men undergoing multigene panel testing for prostate cancer: Emerging implications for personalized prostate cancer genetic evaluation. JCO Precis Oncol (2017), 1–17. doi: 10.1200/po.16.00039 PMC8210976 34164591

[30] Abida W Armenia J Gopalan A Brennan R Walsh M Barron D . Prospective genomic profiling of prostate cancer across disease states reveals germline and somatic alterations that may affect clinical decision making. JCO Precis Oncol (2017) 2017:1–16. doi: 10.1200/po.17.00029 PMC5558263 28825054

[31] de Bono J Mateo J Fizazi K Saad F Shore N Sandhu S . Olaparib for metastatic castration-resistant prostate cancer. New Engl J Med (2020) 382:2091–102. doi: 10.1056/nejmoa1911440 32343890

[32] Albright F Stephenson RA Agarwal N Teerlink CC Lowrance WT Farnham JM . Prostate cancer risk prediction based on complete prostate cancer family history. Prostate (2015) 75:390–8. doi: 10.1002/pros.22925 PMC4293302 25408531

[33] Bratt O Drevin L Akre O Garmo H Stattin P . Family history and probability of prostate cancer, differentiated by risk category: A nationwide population-based study. J Natl Cancer Institute (2016) 108(10):djw110. doi: 10.1093/jnci/djw110 27400876

[34] Jansson F Drevin L Frisell T Stattin P Bratt O Akre O . Concordance of non–low-risk disease among pairs of brothers with prostate cancer. J Clin Oncol (2018) 36:1847–52. doi: 10.1200/JCO.2017.76.6907 29652556

[35] Struewing JP Hartge P Wacholder S Baker SM Berlin M McAdams M . The risk of cancer associated with specific mutations of BRCA1 and BRCA2 among ashkenazi jews. New Engl J Med (1997) 336:1401–8. doi: 10.1056/nejm199705153362001 9145676

[36] Böttcher R Kweldam CF Livingstone J Lalonde E Yamaguchi TN Huang V . Cribriform and intraductal prostate cancer are associated with increased genomic instability and distinct genomic alterations. BMC Cancer (2018) 18(1):8. doi: 10.1186/s12885-017-3976-z 29295717 PMC5751811

[37] Antonarakis ES Shaukat F Isaacsson Velho P Kaur H Shenderov E Pardoll DM . Clinical features and therapeutic outcomes in men with advanced prostate cancer and DNA mismatch repair gene mutations. Eur Urol (2019) 75:378–82. doi: 10.1016/j.eururo.2018.10.009 PMC6377812 30337059

[38] Isaacsson Velho P Silberstein JL Markowski MC Luo J Lotan TL Isaacs WB . Intraductal/ductal histology and lymphovascular invasion are associated with germline DNA-repair gene mutations in prostate cancer. Prostate (2018) 78:401–7. doi: 10.1002/pros.23484 PMC6524639 29368341

[39] Zumsteg ZS Spratt DE Pei I Zhang Z Yamada Y Kollmeier M . A new risk classification system for therapeutic decision making with intermediate-risk prostate cancer patients undergoing dose-escalated external-beam radiation therapy. Eur Urol (2013) 64:895–902. doi: 10.1016/j.eururo.2013.03.033 23541457

[40] Mohler JL Antonarakis ES Armstrong AJ D’Amico AV Davis BJ Dorff T . Prostate cancer, version 2.2019. JNCCN J Natl Compr Cancer Network (2019) 17:479–505. doi: 10.6004/jnccn.2019.0023 31085757

[41] Parker C Castro E Fizazi K Heidenreich A Ost P Procopio G . Prostate cancer: ESMO clinical practice guidelines for diagnosis, treatment and follow-up†. Ann Oncol (2020) 31:1119–34. doi: 10.1016/j.annonc.2020.06.011 32593798

[42] EAU guidelines - uroweb (2022). Available at: https://uroweb.org/guidelines.

[43] Ewing CM Ray AM Lange EM Zuhlke KA Robbins CM Tembe WD . Germline mutations in HOXB13 and prostate-cancer risk. New Engl J Med (2012) 366:141–9. doi: 10.1056/nejmoa1110000 PMC3779870 22236224

[44] Kote-Jarai Z Mikropoulos C Leongamornlert DA Dadaev T Tymrakiewicz M Saunders EJ . Prevalence of the HOXB13 G84E germline mutation in British men and correlation with prostate cancer risk, tumour characteristics and clinical outcomes. Ann Oncol (2015) 26:756–61. doi: 10.1093/annonc/mdv004 25595936

[45] Guedes LB Antonarakis ES Schweizer MT Mirkheshti N Almutairi F Park JC . MSH2 loss in primary prostate cancer. Clin Cancer Res (2017) 23:6863–74. doi: 10.1158/1078-0432.CCR-17-0955 PMC5690834 28790115

[46] Hempelmann JA Lockwood CM Konnick EQ Schweizer MT Antonarakis ES Lotan TL . Microsatellite instability in prostate cancer by PCR or next-generation sequencing. J ImmunoTherapy Cancer (2018) 6(1):29. doi: 10.1186/s40425-018-0341-y PMC5904988 29665853

[47] Middha S Zhang L Nafa K Jayakumaran G Wong D Kim HR . Reliable pan-cancer microsatellite instability assessment by using targeted next-generation sequencing data. JCO Precis Oncol (2017) 2017:1–17. doi: 10.1200/po.17.00084 PMC6130812 30211344

[48] Javle M Curtin NJ . The role of PARP in DNA repair and its therapeutic exploitation. Br J Cancer (2011) 105:1114–22. doi: 10.1038/bjc.2011.382 PMC3208503 21989215

[49] Rose M Burgess JT O’Byrne K Richard DJ Bolderson E . PARP inhibitors: Clinical relevance, mechanisms of action and tumor resistance. Front Cell Dev Biol (2020) 8:564601. doi: 10.3389/fcell.2020.564601 33015058 PMC7509090

[50] Keung M Wu Y Vadgama J . PARP inhibitors as a therapeutic agent for homologous recombination deficiency in breast cancers. J Clin Med (2019) 8:435. doi: 10.3390/jcm8040435 30934991 PMC6517993

[51] Yi T Feng Y Sundaram R Tie Y Zheng H Qian Y . Antitumor efficacy of PARP inhibitors in homologous recombination deficient carcinomas. Int J Cancer (2019) 145:1209–20. doi: 10.1002/ijc.32143 30666631

[52] Min A Im S-A . PARP inhibitors as therapeutics: Beyond modulation of PARylation. Cancers (2020) 12:394. doi: 10.3390/cancers12020394 32046300 PMC7072193

[53] Golan T Hammel P Reni M Van Cutsem E Macarulla T Hall MJ . Maintenance olaparib for germline BRCA -mutated metastatic pancreatic cancer. New Engl J Med (2019) 381:317–27. doi: 10.1056/nejmoa1903387 PMC6810605 31157963

[54] McCabe N Turner NC Lord CJ Kluzek K Białkowska A Swift S . Deficiency in the repair of DNA damage by homologous recombination and sensitivity to Poly(ADP-ribose) polymerase inhibition. Cancer Res (2006) 66:8109–15. doi: 10.1158/0008-5472.CAN-06-0140 16912188

[55] Murai J Huang SYN Das BB Renaud A Zhang Y Doroshow JH . Trapping of PARP1 and PARP2 by clinical PARP inhibitors. Cancer Res (2012) 72:5588–99. doi: 10.1158/0008-5472.CAN-12-2753 PMC3528345 23118055

[56] Lord CJ Ashworth A . BRCAness revisited. Nat Rev Cancer (2016) 16:110–20. doi: 10.1038/nrc.2015.21 26775620

[57] Catalano M Francesco Iannone L Cosso F Generali D Mini E Roviello G . Combining inhibition of immune checkpoints and PARP: Rationale and perspectives in cancer treatment. Expert Opin Ther Targets (2022). doi: 10.1080/14728222.2022.2158813 36519314

[58] Mateo J Carreira S Sandhu S Miranda S Mossop H Perez-Lopez R . DNA-Repair defects and olaparib in metastatic prostate cancer. New Engl J Med (2015) 373:1697–708. doi: 10.1056/nejmoa1506859 PMC5228595 26510020

[59] Astrazeneca. durvalumab granted breakthrough therapy designation by US FDA for treatment of patients with PD-L1 positive urothelial bladder cancer (2016). Available at: https://www.astrazeneca.com/media-centre/press-releases/2016/Lynparza-Olaparib-granted-Breakthrough-Therapy-Designation-by-US-FDA-for-treatment-of-BRCA1-2-or-ATM-gene-mutated-metastatic-Castration-Resistant-Prostate-Cancer-28012016.html.

[60] Mateo J Porta N Bianchini D McGovern U Elliott T Jones R . Olaparib in patients with metastatic castration-resistant prostate cancer with DNA repair gene aberrations (TOPARP-b): a multicentre, open-label, randomised, phase 2 trial. Lancet Oncol (2020) 21:162–74. doi: 10.1016/S1470-2045(19)30684-9 PMC6941219 31806540

[61] US Food and Drug Administration . FDA Approves olaparib for HRR gene-mutated metastatic castration-resistant prostate cancer (2020). Available at: https://www.fda.gov/drugs/resources-information-approved-drugs/fda-approves-olaparib-hrr-gene-mutated-metastatic-castration-resistant-prostate-cancer https://www.fda.gov/drugs/resources-information-approved-drugs/fda-approves-olaparib-hrr-gene-mutated-metastatic-castration-resistant-prostate-cancer.

[62] Abida W Campbell D Patnaik A Sautois B Shapiro J Vogelzang NJ . Preliminary results from the TRITON2 study of rucaparib in patients (pts) with DNA damage repair (DDR)-deficient metastatic castration-resistant prostate cancer (mCRPC): Updated analyses. Ann Oncol (2019) 30:v327–8. doi: 10.1093/annonc/mdz248.003

[63] U.S. Food and Drug Administration . FDA Grants accelerated approval to rucaparib for BRCA-mutated metastatic castration-resistant prostate cancer (2020). Available at: https://www.fda.gov/drugs/resources-information-approved-drugs/fda-grants-accelerated-approval-rucaparib-brca-mutated-metastatic-castration-resistant-prostate https://www.fda.gov/drugs/resources-information-approved-drugs/fda-grants-accelerated-approval-rucaparib-brca-mutated-metastatic-castration-resistant-prostate.

[64] Smith MR Scher HI Sandhu S Efstathiou E Lara PN Yu EY . Niraparib in patients with metastatic castration-resistant prostate cancer and DNA repair gene defects (GALAHAD): a multicentre, open-label, phase 2 trial. Lancet Oncol (2022) 23:362–73. doi: 10.1016/S1470-2045(21)00757-9 PMC9361481 35131040

[65] Dorff TB Fizazi K Laird D Barthélémy P Delva R Maruzzo M . TALAPRO-1: Talazoparib monotherapy in metastatic castration-resistant prostate cancer (mCRPC) with tumor DNA damage response alterations (DDRm)–exploration of germline DDR alteration landscape and potential associations with antitumor activity. J Clin Oncol (2022) 40:157–7. doi: 10.1200/jco.2022.40.6_suppl.157

[66] Palleschi M Tedaldi G Sirico M Virga A Ulivi P De Giorgi U . Moving beyond parp inhibition: Current state and future perspectives in breast cancer. Int J Mol Sci (2021) 22(15):7884. doi: 10.3390/ijms22157884 34360649 PMC8346118

[67] Saad F Armstrong AJ Thiery-Vuillemin A Oya M Loredo E Procopio G . PROpel: Phase III trial of olaparib (ola) and abiraterone (abi) versus placebo (pbo) and abi as first-line (1L) therapy for patients (pts) with metastatic castration-resistant prostate cancer (mCRPC). J Clin Oncol (2022) 40:11–1. doi: 10.1200/jco.2022.40.6_suppl.011

[68] Chi KN Rathkopf DE Smith MR Efstathiou E Attard G Olmos D . Phase 3 MAGNITUDE study: First results of niraparib (NIRA) with abiraterone acetate and prednisone (AAP) as first-line therapy in patients (pts) with metastatic castration-resistant prostate cancer (mCRPC) with and without homologous recombination repair (HRR) gene alterations. J Clin Oncol (2022) 40:12–2. doi: 10.1200/jco.2022.40.6_suppl.012

[69] Vitkin N Nersesian S Siemens DR Koti M . The tumor immune contexture of prostate cancer. Front Immunol (2019) 10:603. doi: 10.3389/fimmu.2019.00603 30984182 PMC6447686

[70] Le DT Uram JN Wang H Bartlett BR Kemberling H Eyring AD . PD-1 blockade in tumors with mismatch-repair deficiency. New Engl J Med (2015) 372:2509–20. doi: 10.1056/nejmoa1500596 PMC4481136 26028255

[71] Marabelle A Le DT Ascierto PA Di Giacomo AM de Jesus-Acosta A Delord JP . Efficacy of pembrolizumab in patients with noncolorectal high microsatellite instability/mismatch repair–deficient cancer: Results from the phase II KEYNOTE-158 study. J Clin Oncol (2020) 38:1–10. doi: 10.1200/JCO.19.02105 31682550 PMC8184060

[72] Kwon ED Drake CG Scher HI Fizazi K Bossi A Van den Eertwegh AJM . Ipilimumab versus placebo after radiotherapy in patients with metastatic castration-resistant prostate cancer that had progressed after docetaxel chemotherapy (CA184-043): A multicentre, randomised, double-blind, phase 3 trial. Lancet Oncol (2014) 15:700–12. doi: 10.1016/S1470-2045(14)70189-5 PMC4418935 24831977

[73] Beer TM Kwon ED Drake CG Fizazi K Logothetis C Gravis G . Randomized, double-blind, phase III trial of ipilimumab versus placebo in asymptomatic or minimally symptomatic patients with metastatic chemotherapy-naive castration-resistant prostate cancer. J Clin Oncol (2017) 35:40–7. doi: 10.1200/JCO.2016.69.1584 28034081

[74] Antonarakis ES Piulats JM Gross-Goupil M Goh J Ojamaa K Hoimes CJ . Pembrolizumab for treatment-refractory metastatic castration-resistant prostate cancer: Multicohort, open-label phase II KEYNOTE-199 study. J Clin Oncol (2020) 38(5):395–405. doi: 10.1200/JCO.19.01638 31774688 PMC7186583

[75] Sharma P Pachynski RK Narayan V Fléchon A Gravis G Galsky MD . Nivolumab plus ipilimumab for metastatic castration-resistant prostate cancer: Preliminary analysis of patients in the CheckMate 650 trial. Cancer Cell (2020) 38:489–499.e3. doi: 10.1016/j.ccell.2020.08.007 32916128

[76] Schweizer MT Cheng HH Tretiakova MS Vakar-Lopez F Klemfuss N Konnick EQ . Mismatch repair deficiency may be common in ductal adenocarcinoma of the prostate. Oncotarget (2016) 7:82504–10. doi: 10.18632/oncotarget.12697 PMC5347709 27756888

[77] Venkatachalam S McFarland TR Agarwal N Swami U . Immune checkpoint inhibitors in prostate cancer. Cancers (2021) 13(9):2187. doi: 10.3390/cancers13092187 34063238 PMC8125096

[78] Capoluongo E Ellison G López-Guerrero JA Penault-Llorca F Ligtenberg MJL Banerjee S . Guidance statement on BRCA1/2 tumor testing in ovarian cancer patients. Semin Oncol (2017) 44:187–97. doi: 10.1053/j.seminoncol.2017.08.004 29248130

[79] Boutros PC Fraser M Harding NJ De Borja R Trudel D Lalonde E . Spatial genomic heterogeneity within localized, multifocal prostate cancer. Nat Genet (2015) 47:736–45. doi: 10.1038/ng.3315 26005866

[80] Ellison G Ahdesmäki M Luke S Waring PM Wallace A Wright R . An evaluation of the challenges to developing tumor BRCA1 and BRCA2 testing methodologies for clinical practice. Hum Mutat (2018) 39:394–405. doi: 10.1002/humu.23375 29215764 PMC5838520

[81] Malapelle U Parente P Pepe F De Luca C Cerino P Covelli C . Impact of pre-analytical factors on MSI test accuracy in mucinous colorectal adenocarcinoma: A multi-assay concordance study. Cells (2020) 9(9):2019. doi: 10.3390/cells9092019 32887373 PMC7565496

[82] Shia J Tang LH Vakiani E Guillem JG Stadler ZK Soslow RA . Immunohistochemistry as first-line screening for detecting colorectal cancer patients at risk for hereditary nonpolyposis colorectal cancer syndrome: A 2-antibody panel may be as predictive as a 4-antibody panel. Am J Surg Pathol (2009) 33:1639–45. doi: 10.1097/PAS.0b013e3181b15aa2 19701074

[83] Raffone A Travaglino A Cerbone M Gencarelli A Mollo A Insabato L . Diagnostic accuracy of immunohistochemistry for mismatch repair proteins as surrogate of microsatellite instability molecular testing in endometrial cancer. Pathol Oncol Res (2020) 26:1417–27. doi: 10.1007/s12253-020-00811-5 32377987

[84] Cicek MS Lindor NM Gallinger S Bapat B Hopper JL Jenkins MA . Quality assessment and correlation of microsatellite instability and immunohistochemical markers among population- and clinic-based colorectal tumors: Results from the colon cancer family registry. J Mol Diagnostics (2011) 13:271–81. doi: 10.1016/j.jmoldx.2010.12.004 PMC3077724 21497289

[85] Alix-Panabières C Pantel K . Liquid biopsy: From discovery to clinical application. Cancer Discovery (2021) 11:858–73. doi: 10.1158/2159-8290.CD-20-1311 33811121

[86] Rzhevskiy AS Bazaz SR Ding L Kapitannikova A Sayyadi N Campbell D . Rapid and label-free isolation of tumour cells from the urine of patients with localised prostate cancer using inertial microfluidics. Cancers (2020) 12(1):81. doi: 10.3390/cancers12010081 PMC7016827 31905736

[87] Martins I Ribeiro IP Jorge J Gonçalves AC Sarmento-Ribeiro AB Melo JB . Liquid biopsies: Applications for cancer diagnosis and monitoring. Genes (2021) 12:1–20. doi: 10.3390/genes12030349 PMC7997281 33673461

[88] Crocetto F Russo G Di Zazzo E Pisapia P Mirto BF Palmieri A . Liquid biopsy in prostate cancer management–current challenges and future perspectives. Cancers (2022) 14(13):3272. doi: 10.3390/cancers14133272 35805043 PMC9265840

[89] Roviello G Lavacchi D Antonuzzo L Catalano M Mini E . Liquid biopsy in colorectal cancer: No longer young, but not yet old. World J Gastroenterol (2022) 28:1503–7. doi: 10.3748/wjg.v28.i15.1503 PMC9048462 35582130

[90] Paunel-Görgülü A Wacker M El Aita M Hassan S Schlachtenberger G Deppe A . CfDNA correlates with endothelial damage after cardiac surgery with prolonged cardiopulmonary bypass and amplifies NETosis in an intracellular TLR9-independent manner. Sci Rep (2017) 7:17421. doi: 10.1038/s41598-017-17561-1 29234042 PMC5727170

[91] Moss J Magenheim J Neiman D Zemmour H Loyfer N Korach A . Comprehensive human cell-type methylation atlas reveals origins of circulating cell-free DNA in health and disease. Nat Commun (2018) 9:1–12. doi: 10.1038/s41467-018-07466-6 30498206 PMC6265251

[92] Atamaniuk J Vidotto C Tschan H Bachl N Stuhlmeier KM Müller MM . Increased concentrations of cell-free plasma DNA after exhaustive exercise. Clin Chem (2004) 50:1668–70. doi: 10.1373/clinchem.2004.034553 15331502

[93] Bettegowda C Sausen M Leary RJ Kinde I Wang Y Agrawal N . Detection of circulating tumor DNA in early- and late-stage human malignancies. Sci Trans Med (2014) 6(224):224ra24. doi: 10.1126/scitranslmed.3007094 PMC4017867 24553385

[94] Schwarzenbach H Hoon DSB Pantel K . Cell-free nucleic acids as biomarkers in cancer patients. Nat Rev Cancer (2011) 11:426–37. doi: 10.1038/nrc3066 21562580

[95] Gasparrini S Cimadamore A Mazzucchelli R Scarpelli M Massari F Raspollini MR . Pathology and molecular updates in tumors of the prostate: towards a personalized approach. Expert Rev Mol Diagnostics (2017) 17:781–9. doi: 10.1080/14737159.2017.1341314 28598696

[96] Cimadamore A Scarpelli M Santoni M Massari F Tartari F Cerqueti R . Genitourinary tumors: Update on molecular biomarkers for diagnosis, prognosis and prediction of response to therapy. Curr Drug Metab (2019) 20:305–12. doi: 10.2174/1389200220666190225124352 30799789

[97] Cimadamore A Gasparrini S Massari F Santoni M Cheng L Lopez-Beltran A . Emerging molecular technologies in renal cell carcinoma: Liquid biopsy. Cancers (2019) 11(2):196. doi: 10.3390/cancers11020196 30736478 PMC6407029

[98] Montironi R Santoni M Cimadamore A Lopez-Beltran A Cheng L . Editorial: Emerging biomarkers in genitourinary tumors. Front Oncol (2019) 9:326. doi: 10.3389/fonc.2019.00326 31106155 PMC6498886

[99] DNA Fragments in the blood plasma of cancer patients: quantitations and evidence for their origin from apoptotic and necrotic cells (2022). Available at: https://ncbi.nlm.nih.gov/11245480/. 11245480

[100] Zocco D Bernardi S Novelli M Astrua C Fava P Zarovni N . Isolation of extracellular vesicles improves the detection of mutant DNA from plasma of metastatic melanoma patients. Sci Rep (2020) 10(1):15745. doi: 10.1038/s41598-020-72834-6 32978468 PMC7519075

[101] Sacher AG Paweletz C Dahlberg SE Alden RS O’Connell A Feeney N . Prospective validation of rapid plasma genotyping for the detection of EGFR and kras mutations in advanced lung cancer. JAMA Oncol (2016) 2:1014–22. doi: 10.1001/jamaoncol.2016.0173 PMC4982795 27055085

[102] Rumford M Lythgoe M McNeish I Gabra H Tookman L Rahman N . Oncologist-led BRCA “mainstreaming” in the ovarian cancer clinic: A study of 255 patients and its impact on their management. Sci Rep (2020) 10:3390. doi: 10.1038/s41598-020-60149-5 32098980 PMC7042365

[103] Volckmar AL Sültmann H Riediger A Fioretos T Schirmacher P Endris V . A field guide for cancer diagnostics using cell-free DNA: From principles to practice and clinical applications. Genes Chromosomes Cancer (2018) 57:123–39. doi: 10.1002/gcc.22517 29205637

[104] Fiala C Diamandis EP . Utility of circulating tumor DNA in cancer diagnostics with emphasis on early detection. BMC Med (2018) 16:166. doi: 10.1186/s12916-018-1157-9 30285732 PMC6167864

[105] Ossandon MR Agrawal L Bernhard EJ Conley BA Dey SM Divi RL . Circulating tumor DNA assays in clinical cancer research. J Natl Cancer Institute (2018) 110:929–34. doi: 10.1093/jnci/djy105 PMC6136923 29931312

[106] Goodall J Mateo J Yuan W Mossop H Porta N Miranda S . Circulating cell-free DNA to guide prostate cancer treatment with PARP inhibition. Cancer Discovery (2017) 7:1006–17. doi: 10.1158/2159-8290.CD-17-0261 PMC6143169 28450425

[107] Vandekerkhove G Struss WJ Annala M Kallio HML Khalaf D Warner EW . Circulating tumor DNA abundance and potential utility in *De novo* metastatic prostate cancer. Eur Urol (2019) 75:667–75. doi: 10.1016/j.eururo.2018.12.042 30638634

[108] Montironi R Cheng L Scarpelli M Cimadamore A Montorsi F Lopez-Beltran A . Re: Gillian vandekerkhove, Werner j. struss, matti annala, et al. circulating tumor DNA abundance and potential utility in *De novo* metastatic prostate cancer. Eur Urol (2019) 75:667–75. doi: 10.1016/j.eururo.2019.05.035 31176624

[109] Jaiswal S Fontanillas P Flannick J Manning A Grauman PV Mar BG . Age-related clonal hematopoiesis associated with adverse outcomes. New Engl J Med (2014) 371:2488–98. doi: 10.1056/nejmoa1408617 PMC4306669 25426837

[110] Hu Y Ulrich BC Supplee J Kuang Y Lizotte PH Feeney NB . False-positive plasma genotyping due to clonal hematopoiesis. Clin Cancer Res (2018) 24:4437–43. doi: 10.1158/1078-0432.CCR-18-0143 29567812

[111] Cimadamore A Cheng L Massari F Santoni M Pepi L Franzese C . Circulating tumor dna testing for homology recombination repair genes in prostate cancer: From the lab to the clinic. Int J Mol Sci (2021) 22(11):5522. doi: 10.3390/ijms22115522 34073818 PMC8197269

[112] Roma C Sacco A Forgione L Esposito Abate R Lambiase M Dotolo S . Low impact of clonal hematopoiesis on the determination of RAS mutations by cell-free DNA testing in routine clinical diagnostics. Diagnostics (2022) 12(8):1956. doi: 10.3390/diagnostics12081956 36010306 PMC9406879

[113] Chan HT Chin YM Nakamura Y Low S-K . Clonal hematopoiesis in liquid biopsy: From biological noise to valuable clinical implications. Cancers (2020) 12:2277. doi: 10.3390/cancers12082277 32823942 PMC7463455

[114] Yu F Makrigiorgos A Leong KW Makrigiorgos GM . Sensitive detection of microsatellite instability in tissues and liquid biopsies: Recent developments and updates. Comput Struct Biotechnol J (2021) 19:4931–40. doi: 10.1016/j.csbj.2021.08.037 PMC8433064 34527197

[115] De Bono J Sweeney C Bracarda S Sternberg CN Chi KN Olmos D . PI3K/AKT pathway biomarkers analysis from the phase III IPATential150 trial of ipatasertib plus abiraterone in metastatic castration-resistant prostate cancer.

[116] Phase A III . Randomized, double-blind, placebo-controlled, multicenter trial testing ipatasertib plus abiraterone plus Prednisone/Prednisolone, relative to placebo plus abiraterone plus Prednisone/Prednisolone in adult Male patients with asymptomatic or mildly symptomatic, previously untreated, metastatic castrate-resistant prostate cancer - AdisInsight (2022). Available at: https://adisinsight.springer.com/trials/700282562.

[117] Abida W Bryce AH Vogelzang NJ Amato RJ Percent I Shapiro JD . Preliminary results from TRITON2: A phase II study of rucaparib in patients (pts) with metastatic castration-resistant prostate cancer (mCRPC) associated with homologous recombination repair (HRR) gene alterations. Ann Oncol (2018) 29:viii272. doi: 10.1093/annonc/mdy284.002

[118] Cimadamore A Scarpelli M Raspollini MR Doria A Galosi AB Massari F . Prostate cancer pathology: What has changed in the last 5 years. Urologia J (2020) 87:3–10. doi: 10.1177/0391560319876821 31545701

[119] Tukachinsky H Madison RW Chung JH Gjoerup OV Severson EA Dennis L . Genomic analysis of circulating tumor DNA in 3,334 patients with advanced prostate cancer identifies targetable BRCA alterations and AR resistance mechanisms. Clin Cancer Res (2021) 27:3094–105. doi: 10.1158/1078-0432.CCR-20-4805 PMC9295199 33558422

[120] Warner E Herberts C Fu S Yip S Wong A Wang G . BRCA2, ATM, and CDK12 defects differentially shape prostate tumor driver genomics and clinical aggression. Clin Cancer Res (2021) 27:1650–62. doi: 10.1158/1078-0432.CCR-20-3708 33414135

[121] Stopsack KH Nandakumar S Wibmer AG Haywood S Weg ES Barnett ES . Oncogenic genomic alterations, clinical phenotypes, and outcomes in metastatic castration-sensitive prostate cancer. Clin Cancer Res (2020) 26:3230–8. doi: 10.1158/1078-0432.CCR-20-0168 PMC7334067 32220891

[122] Schweizer MT Sivakumar S Tukachinsky H Coleman I De Sarkar N Yu EY . Concordance of DNA repair gene mutations in paired primary prostate cancer samples and metastatic tissue or cell-free DNA. JAMA Oncol (2021) 7:1378–82. doi: 10.1001/jamaoncol.2021.2350 PMC8446811 34086042

[123] Wyatt AW Annala M Aggarwal R Beja K Feng F Youngren J . Concordance of circulating tumor DNA and matched metastatic tissue biopsy in prostate cancer. J Natl Cancer Institute (2017) 109(12):djx118. doi: 10.1093/jnci/djx118 PMC6440274 29206995

[124] Chung JH Dewal N Sokol E Mathew P Whitehead R Millis SZ . Prospective comprehensive genomic profiling of primary and metastatic prostate tumors. JCO Precis Oncol (2019), 1–23. doi: 10.1200/po.18.00283 PMC6583915 31218271

[125] Hussain M Corcoran C Sibilla C Fizazi K Saad F Shore N . Tumor genomic testing for >4,000 men with metastatic castration-resistant prostate cancer in the phase III trial PROfound (Olaparib). Clin Cancer Res (2022) 28:1518–30. doi: 10.1158/1078-0432.CCR-21-3940 35091440

[126] Georgiadis A Durham JN Keefer LA Bartlett BR Zielonka M Murphy D . Noninvasive detection of microsatellite instabilit and high tumor mutation burden in cancer patients treated with PD-1 blockade. Clin Cancer Res (2019) 25:7024–34. doi: 10.1158/1078-0432.CCR-19-1372 PMC6892397 31506389

[127] Willis J Lefterova MI Artyomenko A Kasi PM Nakamura Y Mody K . Validation of microsatellite instability detection using a comprehensive plasma-based genotyping panel. Clin Cancer Res (2019) 25:7035–45. doi: 10.1158/1078-0432.CCR-19-1324 31383735

[128] Nakamura Y Okamoto W Kato T Esaki T Kato K Komatsu Y . Circulating tumor DNA-guided treatment with pertuzumab plus trastuzumab for HER2-amplified metastatic colorectal cancer: A phase 2 trial. Nat Med (2021) 27:1899–903. doi: 10.1038/s41591-021-01553-w PMC8604726 34764486

[129] Barata P Agarwal N Nussenzveig R Gerendash B Jaeger E Hatton W . Clinical activity of pembrolizumab in metastatic prostate cancer with microsatellite instability high (MSI-h) detected by circulating tumor DNA. J ImmunoTherapy Cancer (2020) 8(2):e001065. doi: 10.1136/jitc-2020-001065 PMC7422632 32788235

[130] Moss EL Gorsia DN Collins A Sandhu P Foreman N Gore A . Utility of circulating tumor DNA for detection and monitoring of endometrial cancer recurrence and progression. Cancers (2020) 12:1–13. doi: 10.3390/cancers12082231 PMC7463944 32785174

[131] Carter HB Helfand B Mamawala M Wu Y Landis P Yu H . Germline mutations in ATM and BRCA1/2 are associated with grade reclassification in men on active surveillance for prostate Cancer(Figure presented.). Eur Urol (2019) 75:743–9. doi: 10.1016/j.eururo.2018.09.021 PMC6699614 30309687

[132] Castro E Goh C Olmos D Saunders E Leongamornlert D Tymrakiewicz M . Germline BRCA mutations are associated with higher risk of nodal involvement, distant metastasis, and poor survival outcomes in prostate cancer. J Clin Oncol (2013) 31:1748–57. doi: 10.1200/JCO.2012.43.1882 PMC3641696 23569316

[133] Annala M Vandekerkhove G Khalaf D Taavitsainen S Beja K Warner EW . Circulating tumor DNA genomics correlate with resistance to abiraterone and enzalutamide in prostate cancer. Cancer Discovery (2018) 8:444–57. doi: 10.1158/2159-8290.CD-17-0937 29367197

[134] Bednarz-Knoll N Eltze E Semjonow A Brandt B . BRCAness in prostate cancer. Oncotarget (2019) 10:2421–2. doi: 10.18632/oncotarget.26818 PMC6497433 31069005

[135] Omari A Nastały P Stoupiec S Bałabas A Dąbrowska M Bielińska B . Somatic aberrations of BRCA1 gene are associated with ALDH1, EGFR, and tumor progression in prostate cancer. Int J Cancer (2019) 144:607–14. doi: 10.1002/ijc.3190 30265376

